# Gas micronuclei underlying decompression bubbles may explain the influence of oxygen enriched gases during decompression on bubble formation and endothelial function in self-contained underwater breathing apparatus diving

**DOI:** 10.3325/cmj.2019.60.388

**Published:** 2019-08

**Authors:** Ran Arieli

**Affiliations:** 1 The Israel Naval Medical Institute, Israel Defense Forces Medical Corps, Haifa, Israel; 2 Eliachar Research Laboratory, Western Galilee Medical Center, Nahariya, Israel *arieli1940@gmail.com*

**To the Editor:** In a recent study published in the *Croatian Medical Journal*, Šegrt Ribičić et al (1) described the effect of dives using self-contained underwater breathing apparatus on endothelial function (flow-mediated dilatation). Endothelial function was reduced after diving to a depth of 45 m sea water for a bottom time of 20 min when breathing nitrox containing 50% oxygen, but not when breathing air or nitrox containing 99% oxygen. The authors claimed this was the opposite of what they would have expected, it being known that endothelial function is affected by hyperoxia.

We conducted a series of studies on bubble formation using hydrophobic silicon wafers and sheep blood vessels. On the basis of our findings, we proposed that surface nanobubbles were produced from dissolved gas at active hydrophobic spots on the luminal aspect of blood vessels, and that these may be the source of gas micronuclei from which decompression bubbles develop (2). We also suggested that on detachment of a bubble from the blood vessel, it tears off a section of the endothelial membrane and damages the endothelium. The formation of vascular bubbles, therefore, depends both on the availability of gas micronuclei (presumably, only some of the surface nanobubbles become gas micronuclei) and inert gas supersaturation. A study using atomic force microscopy to investigate the influence of various gases on surface nanobubbles found that at 25°C, the diameter of surface nanobubbles composed of nitrogen was 163 nm, and that composed of oxygen was 478 nm (3). It may be that larger surface nanobubbles composed of oxygen will have a greater tendency to transform into gas micronuclei and decompression bubbles.

In the reported study (1), there was no difference in bubble score between air and nitrox 50% dives. A higher nitrogen load in the air dives, and greater availability of gas micronuclei with nitrox 50%, could be the reason for this apparent balance. I would propose that in both of the nitrox dives (50 and 99% oxygen), large nanobubbles were formed at the active hydrophobic spots, but only in nitrox 50% was there also supersaturation of nitrogen, which enabled bubble formation. The detachment of bubbles from blood vessels could have caused the reported endothelial injury in nitrox 50%, but not in nitrox 99%. Thus, when diving with different breathing gas mixtures, the risk of bubble formation should be related not only to the loading of a specific gas, but also to the peculiar properties of the nanobubbles suggested to be the precursors of decompression bubbles.
